# *TaLHY*, a 1R-MYB Transcription Factor, Plays an Important Role in Disease Resistance against Stripe Rust Fungus and Ear Heading in Wheat

**DOI:** 10.1371/journal.pone.0127723

**Published:** 2015-05-26

**Authors:** Zijin Zhang, Jieming Chen, Yongying Su, Hanmei Liu, Yanger Chen, Peigao Luo, Xiaogang Du, Dan Wang, Huaiyu Zhang

**Affiliations:** 1 Biophysics and Immune Engineering Lab, Sichuan Agricultural University, Ya’an, Sichuan, People’s Republic of China; 2 State Key Laboratory of Plant breeding and Genetics, Sichuan Agricultural University, Chengdu, Sichuan, People’s Republic of China; 3 Department of wheat breeding. Puyang Academy of Agricultural Sciences, Puyang, Henan, People’s Republic of China; Institute of Genetics and Developmental Biology, CHINA

## Abstract

LHY (late elongated hypocotyl) is an important gene that regulates and controls biological rhythms in plants. Additionally, LHY is highly expressed in the SSH (suppression subtractive hybridization) cDNA library-induced stripe rust pathogen (CYR32) in our previous research. To identify the function of the LHY gene in disease resistance against stripe rust, we used RACE-PCR technology to clone *TaLHY* in the wheat variety Chuannong19. The cDNA of *TaLHY* is 3085 bp long with an open reading frame of 1947 bp. *TaLHY* is speculated to encode a 70.3 kDa protein of 648 amino acids , which has one typical plant MYB-DNA binding domain; additionally, phylogenetic tree shows that *TaLHY* has the highest homology with LHY of *Brachypodium distachyon*(*BdLHY-like*). Quantitative fluorescence PCR indicates that *TaLHY* has higher expression in the leaf, ear and stem of wheat but lower expression in the root. Infestation of CYR32 can result in up-regulated expression of *TaLHY*, peaking at 72 h. Using VIGS (virus-induced gene silencing) technology to disease-resistant wheat in the fourth leaf stage, plants with silenced *TaLHY* cannot complete their heading stage. Through the compatible interaction with the stripe rust physiological race CYR32, Chuannong 19 loses its immune capability toward the stripe rust pathogen, indicating that *TaLHY* may regulate and participate in the heading of wheat, as well as the defense responses against stripe rust infection.

## Introduction

TFs (transcription factors) are protein molecules that can specifically bind to the cis-acting elements in the promoter region of eukaryotic genes. They can activate or inhibit the transcription of the target genes by interaction with other related proteins[1]. MYB is one of the largest plant transcription factor gene families, all of which have a highly conservative DNA binding domain. The binding domain normally includes 1–3 incompletely repeating sequences (Rs), and each R segment comprises approximately 52 conserved amino acid residues and intervening sequences, folding into a helix-turn-helix (HTH) structure[2]. According to the quantity of repeating R segments, MYB transcription factors can be simply divided into four sub-categories: 1R proteins (R1/R2-MYB), 2R proteins (R2R3-MYB), 3R proteins (R1R2R3-MYB) and 4R proteins (R1R2R2R1/R2-MYB)[3].

MYB transcription factors are widely used in the regulation of secondary metabolism in plant[4], as well as the response to hormone and environmental factors[5]. Moreover, they play an important role in the regulation of plant cell differentiation, organogenesis[6], leaf morphogenesis, and disease resistance[7]. For example, the cotton gene *GhMYBl09* plays a direct role in the formation, elongation and growth of cotton fiber[8]. Over-expression of the *AtMYB24* gene in *Arabidopsis* may result in stunted plant growth and malnutrition of its flower organs[9]. The *AtMYB30* gene is a positive regulation factor of the hypersensitive response (HR) when *Arabidopsis* is upon bacterial infection[10]. Additionally, MYB transcription factors such as AS1 in *Arabidopsis*[11], PHAN in *Antirrhinum*[12], NSPHAN in tobacco[13], and RS2 in corn[14], can all be activated by the induction and protection reaction mechanism of the signaling molecule jasmonic acid, which leads to the over-expression of disease-resistance functional genes, ultimately reducing harm caused by pathogenic bacteria.

LHY is a 1R protein MYB transcription factor (R1/R2-MYB) that, together with CCA1 (circadian clock associated 1) and TOC1 (timing of cab expression l), forms the central oscillator, the central part of the circadian clock in *Arabidopsis*[15]. The protein amino acid sequence of LHY and CCA1 are high homologous, showing similar circadian rhythms and gene function. Numerous reports have demonstrated that LHY is critical to the function of the *Arabidopsis* circadian clock, which plays a major and multi-faceted role in the negative feedback loops that drive the rhythmic expression of the clock genes[16,17]. The LHY promoter is targeted for regulation by other components of the clock oscillator, as well as being regulated by light[18].

Many studies have focused on the function of LHY in the circadian clock in *Arabidopsis*, although it is not clear whether it is involved in the defense response against pathogenic bacteria. Here, we report our cloning and functional studies of a stripe rust pathogen-infested wheat MYB transcription factor that, after bioinformatics analysis, was confirmed to be *TaLHY*. Through the specific expression, circadian rhythm, and expression mechanism of *TaLHY* when it was induced by stripe rust pathogen and hormone, we observed the related influences of *TaLHY* to wheat after VIGS silencing, and assessed the important function of *TaLHY* in the growth and development and the disease-resistant mechanism of wheat.

## Materials and Methods

### Plant materials and inoculation

Two wheat (*Triticum aestivum*) cultivars (Chuannong19 and Mianyang11) and stripe rust Pst pathotype CYR32 were the biological materials used in this study. Chuannong19, which possesses the stripe rust resistance gene *Yr41*, is nearly resistant to CYR32 in different growth stages except the seedling stage[19,20], while Mianyang11 is susceptible to CYR32. After the two varieties grew to the stem elongation stage under conditions of 14 h light/10 h darkness (22°C/10°C), the stripe rust pathogen CYR32 and talc powder were mixed and inoculated onto wheat leaf. The inoculated leaves were sampled at 0, 24, 48, 72 and 96 hours post inoculation(hpi), quickly frozen in liquid nitrogen, and stored at -80°C prior to total RNA extraction. For hormone treatments, wheat seedlings were sprayed with 100 mM methyl jasmonate (MeJA), 100 mM ethylene (ET), 100 mMabscisic acid (ABA) and 100 mM salicylic acid (SA), following Zhang’s method[21]. The leaves were collected for RNA extraction at 0, 1, 2, 6 and 12 h after hormone treatments. In order to invest the circadian rhythms of the expression of *TaLHY*, we chosen the elongation stage of Chuannong 19 and collected the leaves every two hours in one day to extract RNA for Quantitative real-time PCR (qRT-PCR). Each treatment was performed with three independent biological replicates.

### RNA extraction and cDNA synthesis

Total RNA was extracted using TRIzol reagent (TaKaRa, Dalian, China) according to the manufacturer’s instructions. DNase I treatment was applied to remove contaminated genomic DNA. Reverse transcriptase (TAKaRa) was used to synthesize the first strand cDNA according to the manual. qRT-PCR was performed, and SYBR Green I Master Mix (TaKaRa, Japan) was used in a volume of 25 μl with the primers (5’-CCTACTGCTTCCTTTCCCACAAC-3’ and 5’-CTCTCCTTTTCCACTCTCGTCTG-3’) and a CFX96 RT-PCR system (Applied Biosystems). Reactions were set up with the following thermal profile: 1 cycle at 95°C for 10 s, 45 cycles at 95°C for 10 s, 55°C for 20 s, and 68°C for 15 s, followed by 1 cycle at 95°C for 1 min and finally 60°C for 1 min. All qRT-PCR reactions were repeated three times. The relative expression of the gene *TaLHY* was calculated using the 2^-ΔΔ^CT method[22].

### Cloning and sequence analysis of *TaLHY*

Extracts from the Chuannong19 leaves with the cDNA of the cloned *TaLHY* were inoculated with stripe rust pathogen for three days. The SMARTTM RACE cDNA Amplification Kit was used to synthesize 3’-RACE cDNA (primers: 5’-TGTGGTTCCAACACGCCATCAAGTAGTG-3’) and 5’-RACE cDNA (primers: 5’-CTGGTGGGTGTTTCAGAACTGAGACAAC-3’). The resulting PCR products were cloned into the pMD-19T Vector (TaKaRa, Japan) to form positive clones. At least five positive clones were sequenced using an ABI PRISM 3130XL Genetic analyzer (Applied Biosystems, Foster City, CA). DNA sequences were analyzed with DNASTAR (http://www.dnastar.com), BLAST (http://blast.ncbi.nlm.nih.gov/Blast.cgi) and ORF Finder (http://www.ncbi.nlm.nih.gov/gorf/) programs. MEGA4 was used for phylogenetic analysis using the neighbor-joining (NJ) method.

### Functional analysis of *TaLHY* through virus-induced gene silencing

To generate the BSMV:*TaLHY* construct, a 159-bp sequence of *TaLHY* (from 2061 to 2219 nucleotides in the *TaLHY* cDNA sequence) was amplified from Chuannong19 leaves. Next, the fragment was inserted into an antisense orientation into the *NheI* restriction site of RNAγ, resulting in the recombinant construct RNAγ:*TaLHY*. Following a previously described protocol (primers: 5’-gctagcCCTACTGCTTCCTTTCCCACAAC-3’ and 5’-gctagcCTCTCCTTTTCCACTCTCGTCTG-3’)[23], the tripartite cDNA chains of BMSV: *TaLHY*, control virus BMSV:GFP, and BMSV:PDF were separately transcribed into RNA using the mMessagemMachine T7 in vitro transcription kit (Ambion, Austin, TX, U.S.A). The BSMV transcripts were inoculated on the fourth leaf of wheat Chuannong19 by gentle rubbing[24,25]. Next, the fifth leaf was infected by urediniospores of CYR 32 at 10 dpi. The infection types of stripe rust were examined at 15 dpi.

## Results

### Cloning and phylogenetic analyses of *TaLHY*

From the SSH cDNA library of the stripe rust pathogen-induced wheat Chuannong19, we selected the EST sequence whose functional annotation is the MYB transcription factor as the designing primer, and used the rapid amplification of cDNA ends (RACE) method to clone a new wheat MYB transcription factor gene. Sequence analysis indicated that this gene included a complete 1,947-bp open-reading frame (ORF) that encodes a putative protein composed of 648 amino acids with a predicted theoretical molecular weight of 23.13 kDa and an isoeletric point (pI) of 6.34 kDa. Of these 648 amino acids, a 23- to 72-amino acid sequence represented a typical MYB-DNA binding domain. (S1 Fig). BLAST analysis showed that the nucleotide sequence of this gene was highly similar to those of predicted LHY-like proteins from *Brachypodium distachyon* (GenBank accession NO. XM_003573421.1) (83% identity) and *Setar iaitalica* (GenBank accession NO. XM_004972742.1)(79% identity). Thus, we designated the gene as *TaLHY* (GenBank accession NO: HQ222606.1).

**Fig 1:**
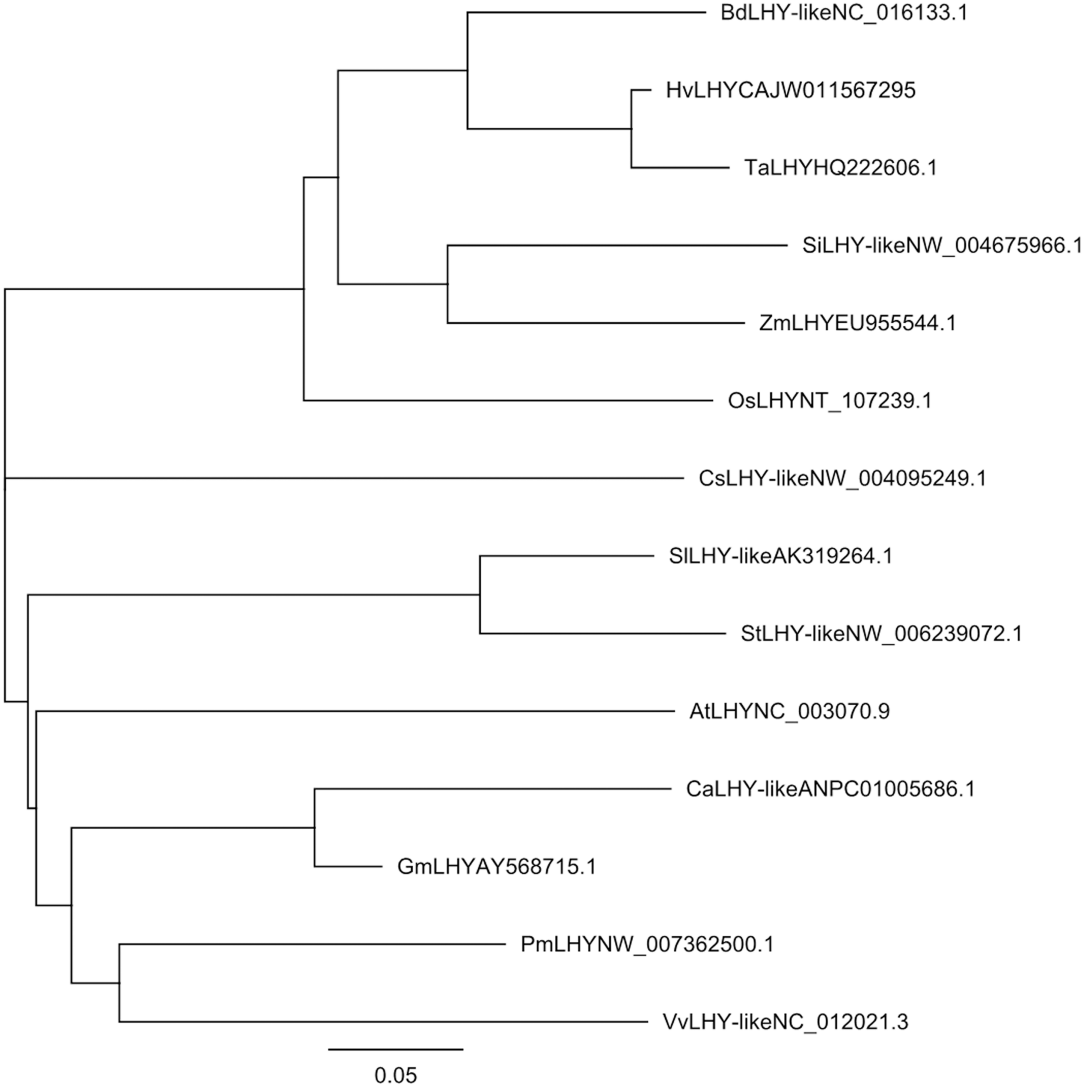
A representative phylogenetic tree of *TaLHY* and selected LHY genes. Five LHY sequences of monocotyledon plant species from *Brachypodium distachyon*, *Hordeum vulgare*, *Oryza Sativa*, *Setaria italica* and *Zea mays*, and eight LHY sequences of dicotyledon plant species from *Solanum lycopersicum*, *Solanum tuberosum*, *Arabidopsis*, *Cicer arietinum*, *Glycine max*, *Prunu smume*, *Vitis vinifera* and *Cucumis sativus* were selected. GeneBank accession numbers are provided after the gene names.

**Fig 2:**
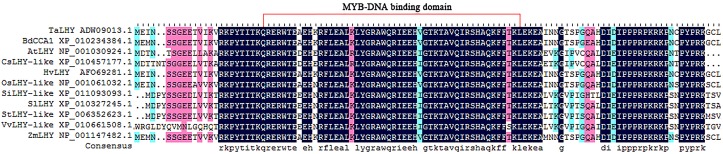
LHY amino acid sequence multiple alignment results in different plants. The conserved MYB-DNA binding domain of different plants is marked by red box.

Phylogenetic analysis revealed the homology relationship among *TaLHY* and related LHY genes in other plant species. Five LHY sequences of monocotyledon plant species and 8 LHY sequences of dicotyledon plant species were downloaded from GenBank to construct a neighbor-joining phylogenetic tree with *TaLHY* (Fig 1). The results suggested that the LHY genes of 6 monocotyledon plant species formed a conservative independent branch, among which *TaLHY* had the closest homology to *HvLHY*. However, the LHY genes of 8 dicotyledon plant species showed a more complicated homology relationship. Additionally, the result of multiple alignment demonstrated that the MYB-DNA binding domain of LHY in different plants are conserved (Fig 2). Among the 50 amino acids length of MYB-DNA binding domain in 11 different plants, 45 amino acids are consistent. Fig 2 showed that the functional domains of TaLHY and other LHY family gene are highly conserved in monocots and dicots.

### Tissue-specific expression and circadian rhythm of *TaLHY*

**Fig 3:**
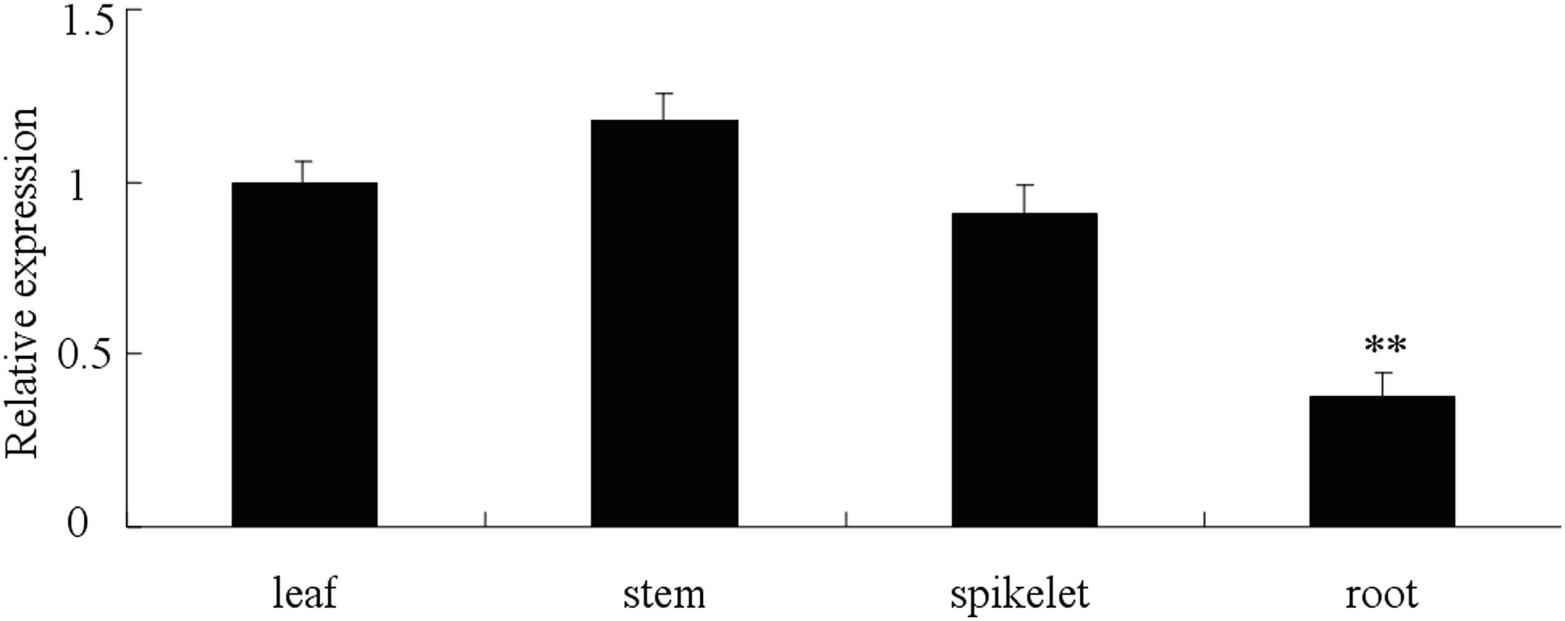
Expression profiles of the *TaLHY* gene in different wheat tissues. The relative expression of *TaLHY* was normalized to the transcript abundances in leaves (as 1). The error bars represent the standard deviation among three biological replicates. The asterisks indicate statistically significant variation calculated using Student’s t-test. (*P<0.05; ** P<0.01).

**Fig 4:**
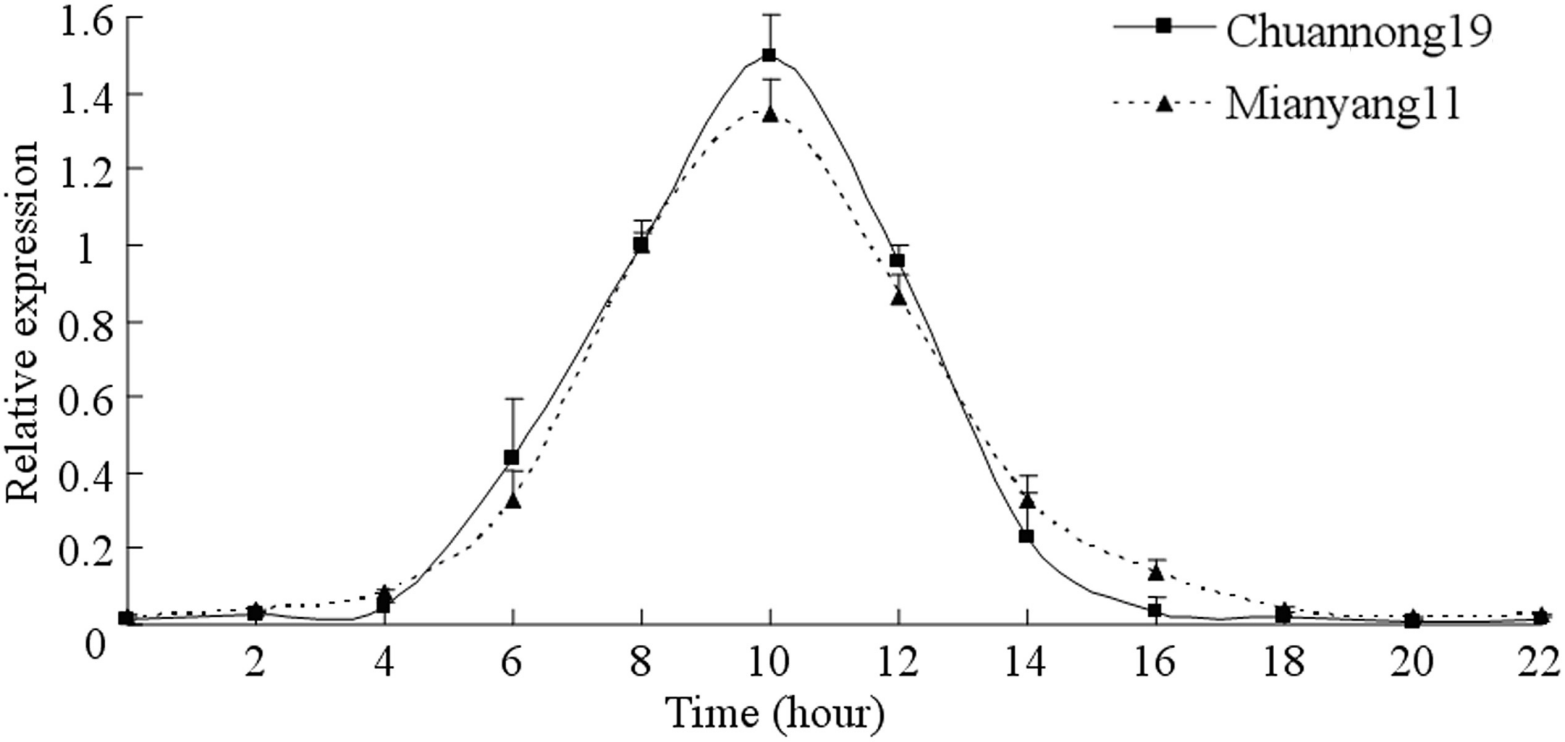
Expression profiles of the *TaLHY* gene in circadian rhythm. The relative expression of *TaLHY* was normalized to the transcript abundances at 8 a.m. (as 1). The error bars represent the standard deviation among three biological replicates. (units: %).

The relative expression value was measured in different tissues in wheat Chuannong19 using qRT-PCR. The results revealed that the expression levels of *TaLHY* were different between root and other tissues. By normalizing the level of the *TaLHY* transcript in leaves as 1,the expression levels in the stem, leaf and spike remained at the same level, with no statistically significant differences. Howere, the relative expression of *TaLHY* in the root of wheat was 50% lower than that in other tissues (Fig 3). As a transcription factor to regulate the biological rhythm of the plant, the expression level of LHY itself also has a circadian rhythm. Using qRT-PCR to measure the circadian rhythm of *TaLHY* in wheat during the jointing stage, we found that the expression of *TaLHY* has a very obvious circadian rhythm. Normalizing the level of the *TaLHY* transcript at 8 a.m. as 1, the relative *TaLHY* expression was very low from 0 to 4 a.m., and from 4 p.m. to 10 p.m.. At 6 a.m., the relative gene expression gradually rises and peaks at 10 a.m. (Fig 4).

### The expression patterns of *TaLHY* are induced by the stripe rust pathogen and exogenous hormone

**Fig 5:**
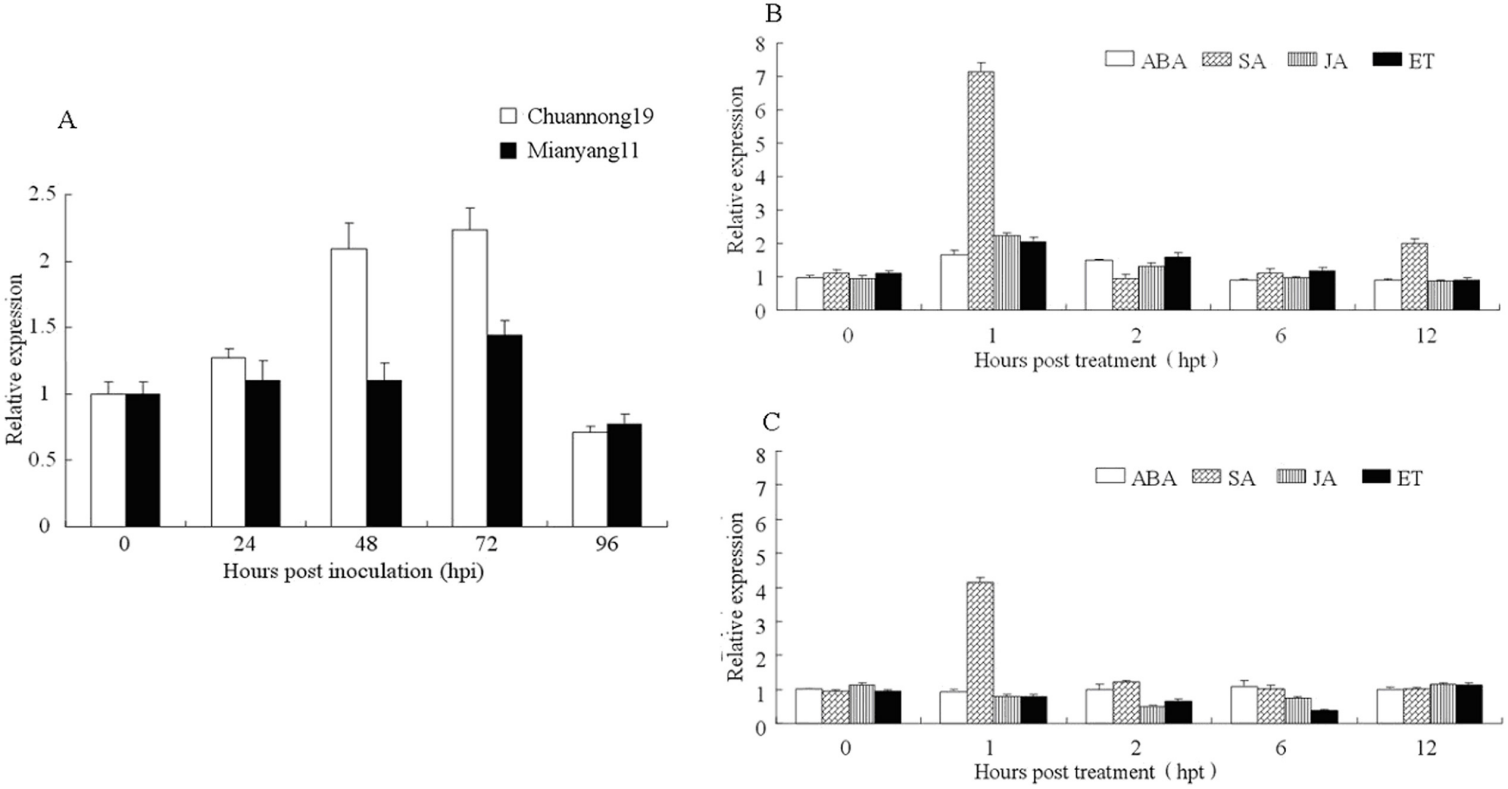
Expression profiles of *TaLHY* in wheat Chuannong19 and Mianyang11 responding to different treatments. A. Response to the stripe rust. B. Response to four exogenous chemicals in wheat Chuannong19. C. Response to four exogenous chemicals in wheat Mianyang11. ABA: abscisic acid; SA: salicylic acid; JA: jasmonate acid; ET: ethylene. Stripe rust pathogen treatment at every time point is normalized at 0 hip, and exogenous hormone treatment at every time point is normalized at the corresponding circadian rhythm transcript abundance. The error bars represent the standard deviation among three biological replicates.

In order to analyze the expression pattern of *TaLHY* induced by CYR32, the leaves of disease-resistant wheat Chuannong19 and susceptible wheat Mianyang11 were inoculated with the stripe rust pathogen race CYR32, respectively. Quantitative fluorescence PCR was used to measure the relative expression of *TaLHY* at 0, 24, 48, 72 and 96 hours post inoculation (hpi). The result showed that, at 24 and 48 hpi, the relative expression of *TaLHY* in wheat Mianyang11 was slightly up-regulated, and peaked obviously at 72 hpi (Fig 5a). However, the relative expression of *TaLHY* in wheat Chuannong19 was significantly up-regulated at 48 and 72 hpi compared with the expression at 0 hpi (p<0.01), which was 2-fold of the relative expression at 0 hpi, and also significantly higher than that in the susceptible wheat at the corresponding time (p<0.01). Thus, in wheat Chuannong19, the stripe rust pathogen infestation can induce *TaLHY* to express remarkably.

As pathogenic bacteria infestation can induce *TaLHY* to express, we further investigate *TaLHY*’s response to signaling molecules, including 4 different exogenous hormones. Abscisic acid (ABA), salicylic acid (SA), jasmonic acid (JA) and ethanol (ET), were selected to treat the leaves of wheat Chuannong19 and wheat Mianyang11 during the jointing stage, and the relative expression of *TaLHY* in the leaves were measured at 5 time points: 0, 1, 2, 6 and 12 hours post treatment (hpt) (Fig 5b and 5c). Due to the transcript of *TaLHY* having an obvious circadian rhythm, to precisely reflect the response of *TaLHY* to induction by these exogenous hormones, we normalized the level of the *TaLHY* transcript at each time point. After treatment with ABA, the *TaLHY* expression level in Chuannong19 at 1 hpi reached a peak that was significant (p<0.05), but the relative expression of *TaLHY* in Mianyang11 treated with ABA was almost not changed. At 1 hpi after treatment with SA, the relative expression levels of *TaLHY* in Chuannong19 and Mianyang11 both reached their peaks and were 7-fold and 4-fold of the expression at 0 hpi, respectively, and the expression was much higher than Chuannong19 and Mianyang11 were treated with other hormones at this time point, with the relative expression of *TaLHY* in Chuannong19 being much higher than that in Mianyang11 (P<0.01). After treatment with JA and ET, the relative expression of *TaLHY* in Chuannong19 was up-regulated, and peaked at 1 hpi, which was 2-fold higher than that at 0 hpi. By contrast, the relative expression of *TaLHY* was down-regulated by these two hormones in Mianyang11.

### Down-regulating the expression of *TaLHY* affects the growth of wheat and resistance to the stripe rust pathogen

**Fig 6:**
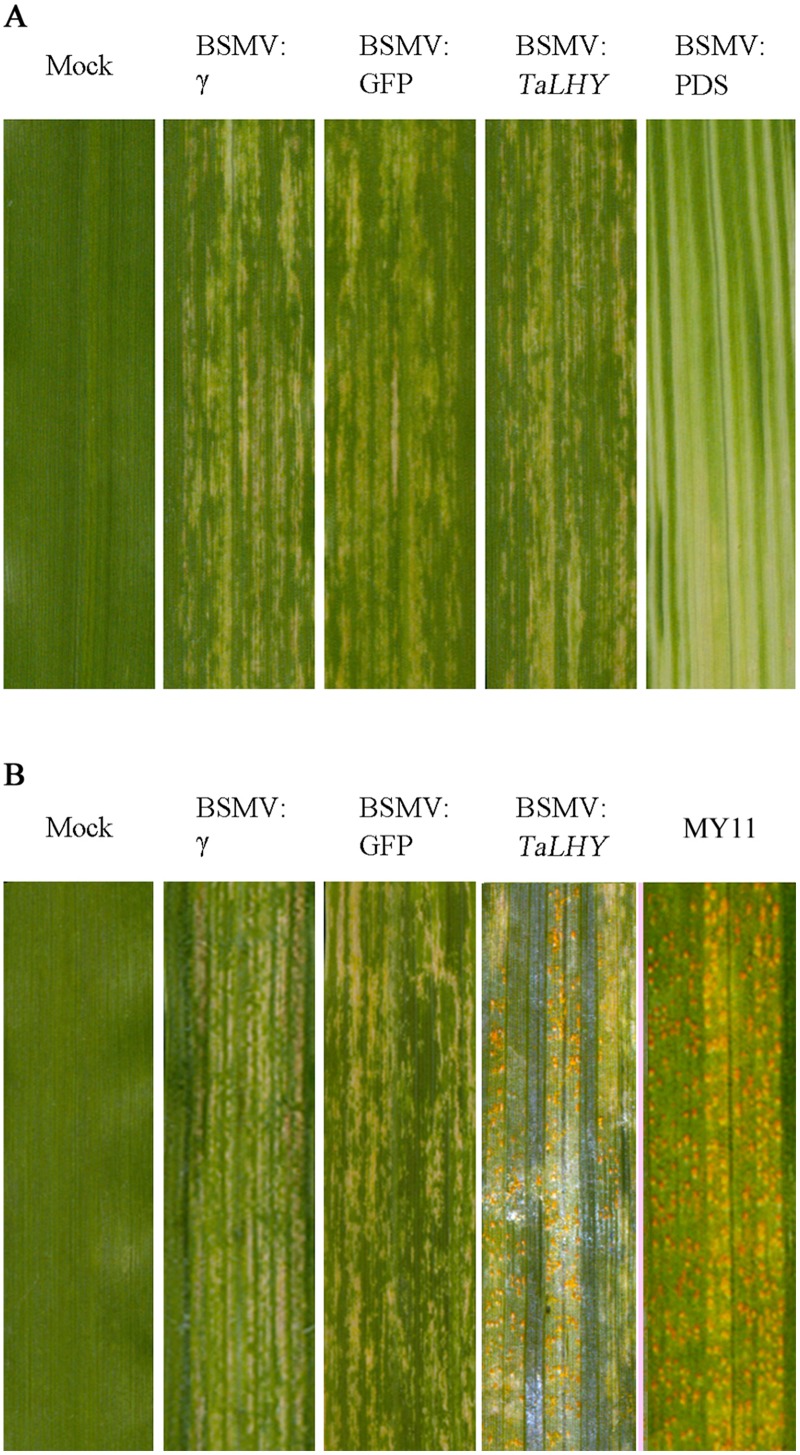
Results of wheat leaves with silenced target genes by VIGS and the leaves of wheat infested by stripe rust after gene silencing. A. Mild chlorotic mosaic symptoms were observed on the leaves inoculated with BSMV: γ、BSMV: *TaLHY*、and BSMV: GFP at 10 dpi. Mock: Chuannong19 leaves treated with buffer. Photobleaching was evident on leaves infected with BSMV: PDS at 15 dpi but not on mock-inoculated leaves. B. Stripe rust infection types of Chuannong19 at 15 dpi with CYR32. Mianyang 11: comparison of disease infection. Typical leaves were photographed at 15 dpi.

**Fig 7:**
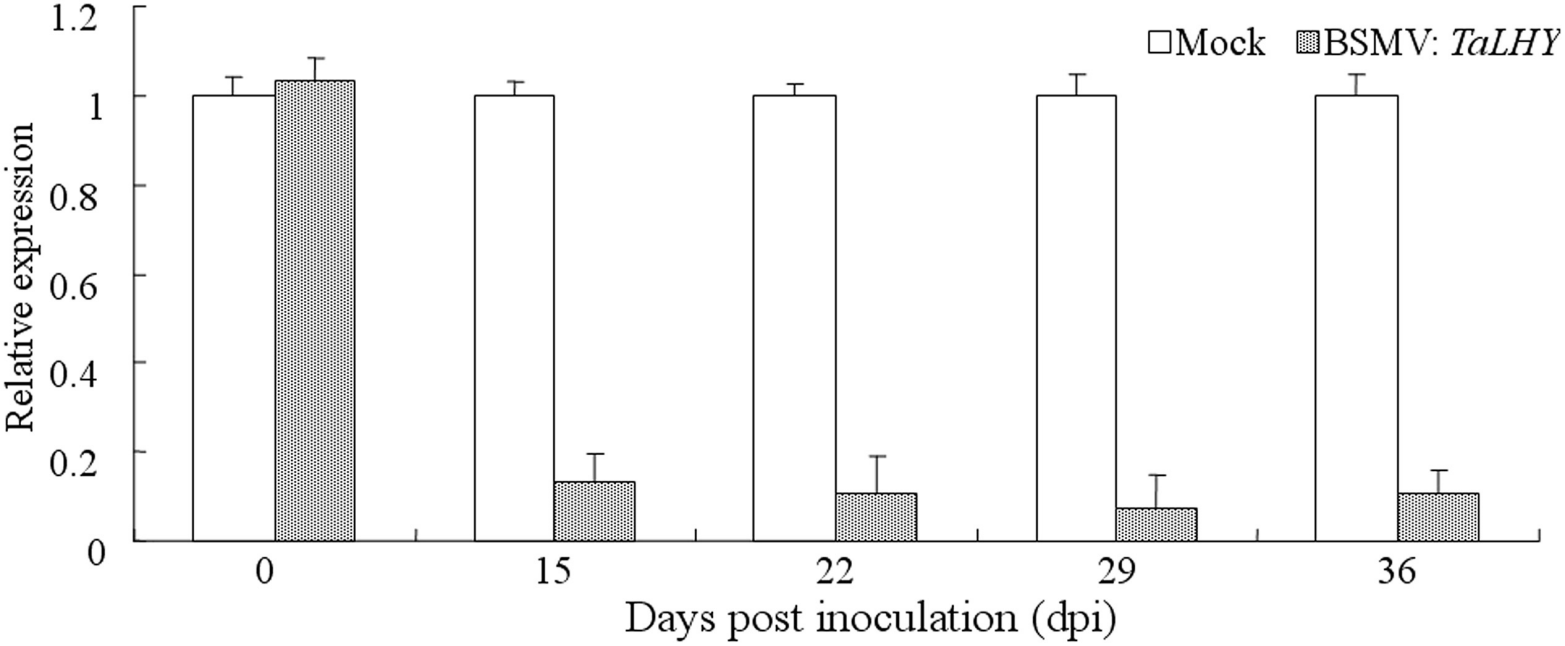
The relative expression of *TaLHY* in wheat Chuannong19 in response to transfection with BSMV:*TaLHY*. The relative expression of *TaLHY* was normalized to the transcript abundances in Mock (as 1). The error bars represent the standard deviation among three biological replicates.

**Fig 8:**
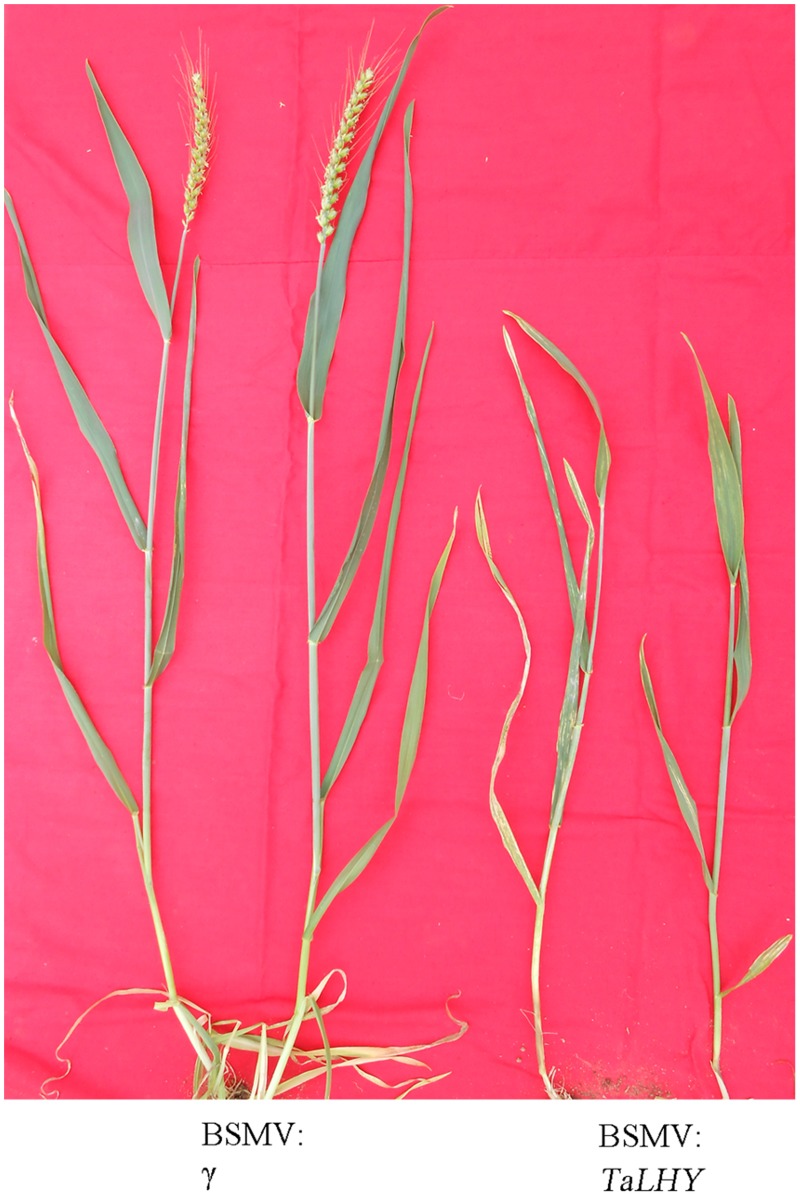
The growth of wheat plants after gene silencing. The left two were control Chuannong19 plants, with normal heading and flowering. The right two were *TaLHY*-silenced plants, unable to enter the heading stage of wheat development.

To study the regulation effects of *TaLHY* on wheat growth and disease resistance, Barley stripe mosaic virus-induced gene silencing (BSMV-VIGS) technology was used in wheat Chuannong19 to knock down *TaLHY*. The silencing vector BSMV:PDS was constructed to contain a 159-bp fragment of *TaLHY*. In addition, the buffer and recombination vectors BSMV: γ、BSMV:PDS and BSMV:GFP used for inoculation in the assay were used as a reference in the study to verify the results of virus infestation. 10 days after virus-activated RNA inoculation of the leaves of all wheat Chuannong19 during the jointing stage, the wheat treated with BSMV:PDS, other the recombined viruses, and buffer (Mock) showed photobleaching, virus spots, and a normal phenotype, respectively (Fig 6a). Using qRT-PCR to test the expression of *TaLHY* and PDS in the *TaLHY*-silenced plant, we discovered that the relative expression levels of the corresponding silencing genes were 15, 22, 29, and 36 dpi, which were significantly lower (P <0.01) than the weight before they were transferred, indicating that genes were silenced (Fig 7). The stripe rust pathogen infestation assay revealed that Mock and BSMV:γ and BSMV:GFP-treated plants can resist to CYR32. Many stripe rust pathogen soruses grown on the leaves of Chuannong 19 when treated with BSMV:*TaLHY*. As a comparison, Mianyang 11 was also infested by stripe rust.(Fig 6b). Additionally, BSMV:*TaLHY*-treated plants stopped growing and remained in the jointing stage, failing to progress to the heading stage (Fig 8).

## Discussion

In this assay, we identified and cloned a MYB transcription factor called *TaLHY*. *TaLHY* has a 3085-bp-long cDNA. By analyzing the amino acid sequence, it was confirmed that there is a highly conservative MYB-DNA binding domain in *TaLHY*, which is an important structure that facilitates the performance of the *TaLHY* gene as a transcription factor. Because of numerous MYB transcription factors and their complex evolutionary relationships, we only selected LHYs from 13 other plant species to analyze the evolutionary relationships between them and *TaLHY*. It was shown that *TaLHY* has a relatively high homology with the LHY protein in monocotyledon plants such as barley, *Brachypodium distachyon* and wheat. Howere, a difference was noted with dicotyledon plants, which indicated that LHY has different adaptive selections between monocotyledon and dicotyledon plants during evolution. Regarding the evolution model of MYB transcription factors, it is generally recognized that 3R-MYB lacks the 1R sequence and results in 2R-MYB[26], or 2R-MYB gains the 1R sequence and forms 3R-MYB[27]. The homology analysis indicates that the evolution process of 1R-MYB itself is diversified and complex.

*TaLHY* has a relatively high expression in the leaf, ear and stem of the wheat resulting from its regulation function in the growth and development of the plant. A large number of reports focusing on the LHY of *Arabidopsis* have shown that LHY not only demonstrates obvious circadian rhythm but restrains and regulates the flowering of the plant together with CCA1, synergistically[28,29]. Furthermore, over-expression of LHY can also destroy the biological clock rhythm of *Arabidopsis*[30]. The assay revealed that the expression of *TaLHY* is influenced by light, indicating that it may accumulates at dawn, peaks in the morning, and maintains a very low expression level at night. When *TaLHY* is being down-regulated, the flowering of wheat becomes influenced and cannot enter the heading stage. This article identifies that the regulation of circadian rhythm and plant growth and development by *TaLHY* gene of the monocotyledon wheat is similar to the function of the *LHY* gene in *Arabidopsis*. Moreover, LHY in *Arabidopsis*, as an upstream regulating factor, shows that the physiological regulation of the plant’s circadian rhythm, flowering, and so on is a very complex process and is also regulated by a series genes such as ELF3 (early flowering 3), PHYA (phytochrome A), PHYB (phytochrome B), GI (gigantea), ZTL (zeitlupe 1), FKF1 (flavin-binding kelch repeat fbox 1) and DET1 (deetiolated 1)[31]. Further research is needed to detail the regulation mechanism of *TaLHY* in the growth and development of wheat.

Similar to the disease-resistance-related MYB transcription factors that have been discovered previously[32], the expression of *TaLHY* is affected by the stripe rust pathogen infestation. However, the stripe rust pathogen infestation can induce the up-regulation of *TaLHY* more obviously in disease-resistant wheat Chuannong19 than that in susceptible wheat Mianyang11, indicating that the effects of pathogenic bacteria infestation on *TaLHY* are varied in disease-resistant and—susceptible wheat species. Currently, it has been found that the MYB transcription factor has a certain broad-spectrum effect on disease resistance, but our results cannot ensure that the *TaLHY* gene has a broad-spectrum effect or specificity on disease resistance. However, when *TaLHY* in wheat Chuannong19 is down-regulated, the plant loses its immune capability toward stripe rust pathogen, proving that *TaLHY* indeed plays a key role as a positive regulation factor as an upstream regulation factor in the disease resistance of wheat.

After pathogenic bacteria infest a plant, hormone-induced plant systemic resistance can result from different treatment pathways, such as following treatment with SA or JA/Et. Our results revealed that the expression of *TaLHY* is closely related to the SA signal transduction pathway; however, regarding the ABA and JA/Et treatment pathways, *TaLHY* expression is obviously varied in disease-resistant and—susceptible wheat species, showing that the transduction pathway of the signal molecule of this transcription factor is diversified. Considering the complexity of a plant’s systemic regulation network, the precise regulation of the target gene needs the interactions between multiple types of transcription factors and signal molecules. This article only defines the key function of *TaLHY* in wheat growth and development and disease resistance, thus the detailed regulation mechanism still requires further investigation.

## Supporting Information

S1 Fig The complete cDNA sequence and deduced amino acid sequence of the *TaLHY* gene. The conserved MYB-DNA binding domain motif is marked by the box.
(TIF)
