# Supplementary figures and images for: TaLHY, a 1R-MYB Transcription Factor, Plays an Important Role in Disease Resistance against Stripe Rust Fungus and Ear Heading in Wheat
Source: PLoS One. 2015 May 26;10(5):e0127723. doi: 10.1371/journal.pone.0127723 (PMC4444181; doi:10.1371/journal.pone.0127723)

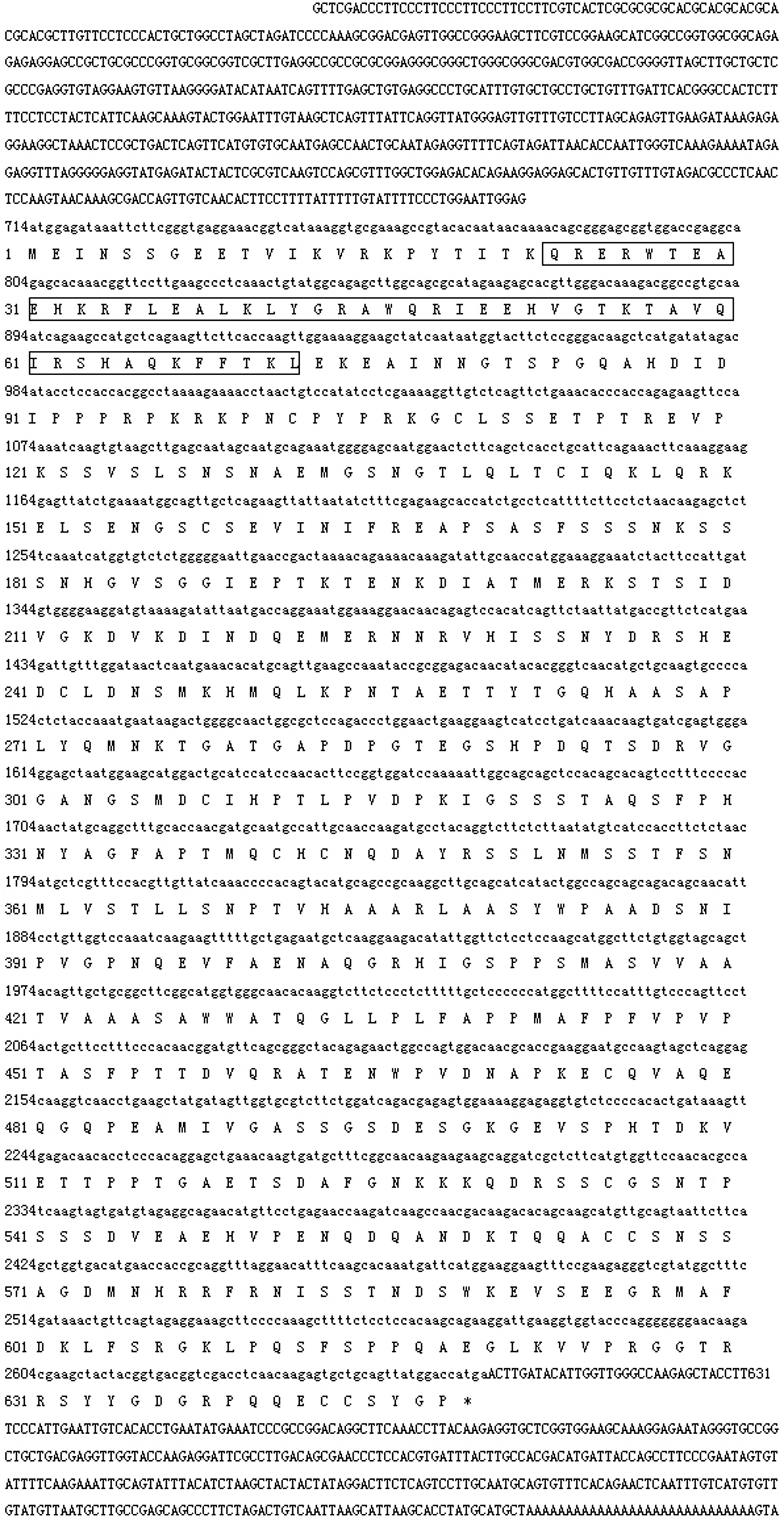

Supplement: S1 Fig — The conserved MYB-DNA binding domain motif is marked by the box. (TIF) [file pone.0127723.s001.tif]
